# The cell wall hydrolase MltG is essential to maintain cell wall homeostasis of *Enterococcus faecalis*

**DOI:** 10.1128/jb.00056-25

**Published:** 2025-06-13

**Authors:** Alexis A. U. Knotek, Christopher J. Kristich

**Affiliations:** 1 Department of Microbiology and Immunology, Center for Infectious Disease Research, Medical College of Wisconsin 735651, Milwaukee, Wisconsin, USA; The Ohio State University, Columbus, Ohio, USA

**Keywords:** *Enterococcus*, peptidoglycan, MltG, cephalosporin resistance, cell wall stress

## Abstract

Infections caused by enterococci are increasingly prevalent and difficult to treat due to multidrug resistance. *Enterococcus faecalis* exhibits intrinsic resistance toward cephalosporins, which inhibit the final step of peptidoglycan (PG) synthesis. Intrinsic resistance requires multiple factors in the PG synthesis pathway and at least two cell-wall-stress signal transduction systems; however, the complete molecular mechanism of enterococcal cephalosporin resistance remains to be elucidated. MltG, a predicted PG hydrolase, is thought to process nascent strands of PG, suggesting that MltG might play an important role in enterococcal cell wall homeostasis and potentially cephalosporin resistance. Here, we demonstrate that enterococcal MltG cleaves nascent PG. An *E. faecalis* mutant lacking MltG exhibits several related phenotypes in the absence of exogenous stress: a marked growth defect, a loss of cell wall integrity, a reduction in PG synthesis, and activation of two cell-wall-stress signal transduction systems that drive elevated cephalosporin resistance. Together, these results are consistent with the model that MltG promotes proper cell wall homeostasis in *E. faecalis,* and further reveal that the enzymatic activity of MltG is not necessary for it to perform this function—instead, the LysM (putative PG-binding) domain of MltG plays the critical role. Nevertheless, the enzymatic activity of MltG does impact cephalosporin resistance, because a catalytically inactive MltG variant leads to elevated resistance. Collectively, our findings represent the first description of MltG function in *E. faecalis* and point to at least two distinct roles for MltG in PG homeostasis and cephalosporin resistance.

**IMPORTANCE:**

*Enterococcus faecalis* is an opportunistic pathogen that colonizes the human gut microbiome. Infections caused by *E. faecalis* are increasingly prevalent and difficult to treat due to the multidrug resistance exhibited toward common clinical antibiotics. A thorough understanding of the mechanisms used by *E. faecalis* to maintain cell wall homeostasis will serve as a foundation for future development of new therapeutics that disable enterococcal resistance to cell-wall-active antibiotics and may reveal new vulnerabilities that could be exploited by novel antimicrobials. Here, we demonstrate that the MltG peptidoglycan hydrolase is essential for enterococcal cell wall homeostasis, but that the enzymatic activity of MltG is not required for this role. Instead, the enzymatic activity of MltG impacts intrinsic resistance toward cephalosporins.

## INTRODUCTION

*Enterococcus faecalis* is a Gram-positive bacterium that is a commensal within the healthy human gut microbiome (1). However, *E. faecalis* exhibits both intrinsic and acquired resistance toward many clinical antibiotics, including cephalosporins (2–4). Due to these antibiotic-resistant properties, *E. faecalis* can persist following antibiotic treatments while other susceptible bacteria are diminished within the gastrointestinal tract (GIT), allowing *E. faecalis* to proliferate within the GIT and escape from the gut to establish infections (5–8). Prior treatment with cephalosporins is therefore a risk factor for the establishment of enterococcal infections, which are commonly healthcare-acquired (5, 9–11). In fact, enterococci are one of the leading causes of healthcare-acquired infections (12–14). Unfortunately, these infections are also increasingly difficult to treat as the prevalence of antibiotic-resistant enterococci increases (15–17). Therefore, there is an urgent need to understand the antibiotic resistance mechanisms of *E. faecalis*.

*E. faecalis* exhibits intrinsic resistance toward cephalosporins that inhibit the final step of peptidoglycan (PG) synthesis, transpeptidation, normally carried out by penicillin-binding proteins (PBPs) (18). This intrinsic cephalosporin resistance is in part mediated by “low-reactivity” PBPs, which can perform their transpeptidase activity in the presence of cephalosporins (19–21), as well as other factors involved in PG synthesis (22–25). The intrinsic cephalosporin resistance of *E. faecalis* is also driven by activation of the cell wall stress signaling systems, IreK and CroRS (26, 27). IreK is a ser/thr kinase that senses cell wall stress, leading to its autophosphorylation and activation of kinase activity to phosphorylate downstream substrates (28–30). CroRS is a two-component signal transduction system that also senses cell wall stress (CroS), leading to phosphorylation of the response regulator (CroR), which subsequently binds DNA as a transcription factor to promote expression of genes within its regulon, including some low-reactivity PBPs (31, 32) such as Pbp4 and PbpA.

PG homeostasis is complex, requiring not only the PG synthases but also PG hydrolytic enzymes to ensure proper integration of newly synthesized PG strands into the existing PG matrix. One such hydrolase thought to be important for maintaining PG homeostasis is MltG. MltG homologs exhibit lytic transglycosylase activity on PG, with a preference for nascent, uncrosslinked PG strands (33–36). This catalytic activity requires a key, conserved glutamate residue within the extracellular YceG domain (33–37). MltG also contains an extracellular LysM domain, with a putative PG binding function, which is essential for determining the length of PG strands produced by MltG homologs *in vitro* and necessary for functional complementation *in vivo* (34, 38). In addition, MltG homologs are known to interact with various PBPs in different bacterial species (33, 36, 38). The prevailing model suggests that MltG functions as a terminase for the synthesis of PG strands (terminating transglycosylation by PG synthases when PG polymers have achieved the appropriate length), thereby priming the nascent PG strands for crosslinking by PBPs and integration into the mature cell wall.

The function of MltG in enterococci has not been investigated thus far. Given the proposed role of MltG in PG homeostasis and the requirement for multiple PG synthesis factors for enterococcal cephalosporin resistance, we hypothesized that MltG may influence the intrinsic cephalosporin resistance exhibited by *E. faecalis*. Here, we demonstrate that MltG, and specifically its LysM domain, is essential to the maintenance of enterococcal cell wall homeostasis and describe a novel mechanism to activate the enterococcal cell wall stress signaling systems to drive resistance toward cephalosporins, providing new insights into enterococcal cell wall homeostasis and intrinsic cephalosporin resistance.

## RESULTS

### MltG of *E. faecalis* cleaves nascent peptidoglycan

**Fig 1:**
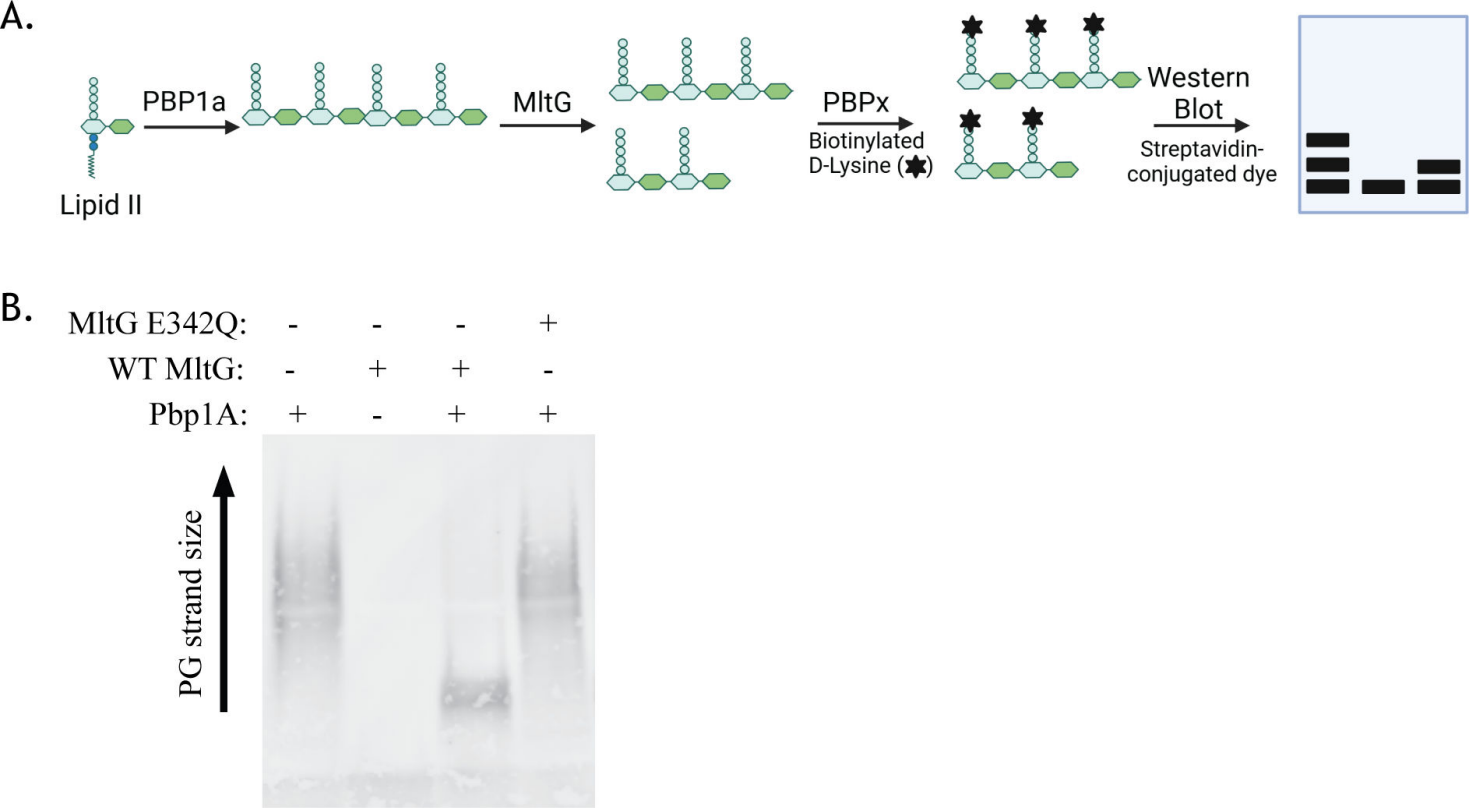
Enterococcal MltG cleaves nascent PG products *in vitro*. (**A**) Schematic of the catalytic activity assay used to visualize PG cleavage by MltG. Briefly, purified Pbp1A was incubated with Lipid II, followed by the addition of either wild-type (WT) MltG or MltG E342Q. PG products were then labeled with Biotinylated D-Lysine and run on 4%–20% gradient SDS-PAGE gel, and PG was detected with IR-streptavidin. (**B**) SDS-PAGE analysis of reactions with the indicated composition.

Homologs of MltG have been demonstrated to cleave PG strands, with a preference for nascent uncrosslinked PG (33–35). To determine whether MltG of *E. faecalis* also cleaves nascent PG, we modified a previously described *in vitro* assay outlined in Fig. 1A (34). PG strands were first synthesized from the Lipid II precursor by the PG synthase Pbp1A. To prevent any potential influence of MltG on the synthesis of PG by Pbp1A, PG synthesis by Pbp1A was performed first, and then MltG was added to the reactions after termination of the initial PG strand synthesis. PG products were then labeled and separated by SDS-PAGE (Fig. 1B). Reactions with no Pbp1A did not synthesize PG products from Lipid II, confirming the absence of any PG synthase contamination in the MltG preparations. Pbp1A-synthesized PG that was not treated with MltG contained long strands of PG, indicated by the high molecular weight smear of products. Pbp1A-synthesized PG that was treated with wild-type MltG led to a significant decrease in the size of PG products compared to those produced by Pbp1A alone, demonstrating that MltG can cleave the PG to smaller products. In addition, a variant of MltG with a mutation of the conserved catalytic glutamate residue (Fig. S1), MltG E342Q (a substitution previously demonstrated to impair catalysis by MltG homologs (33, 35–37), did not cleave PG to smaller products, indicating that the catalytic activity of MltG is required. Together, these data indicate that *E. faecalis* MltG is capable of cleaving nascent strands of PG following transglycosylation, consistent with the known function of MltG homologs from other bacterial species (33, 35, 37, 38).

### Deletion of *mltG* impairs growth

**Fig 2:**
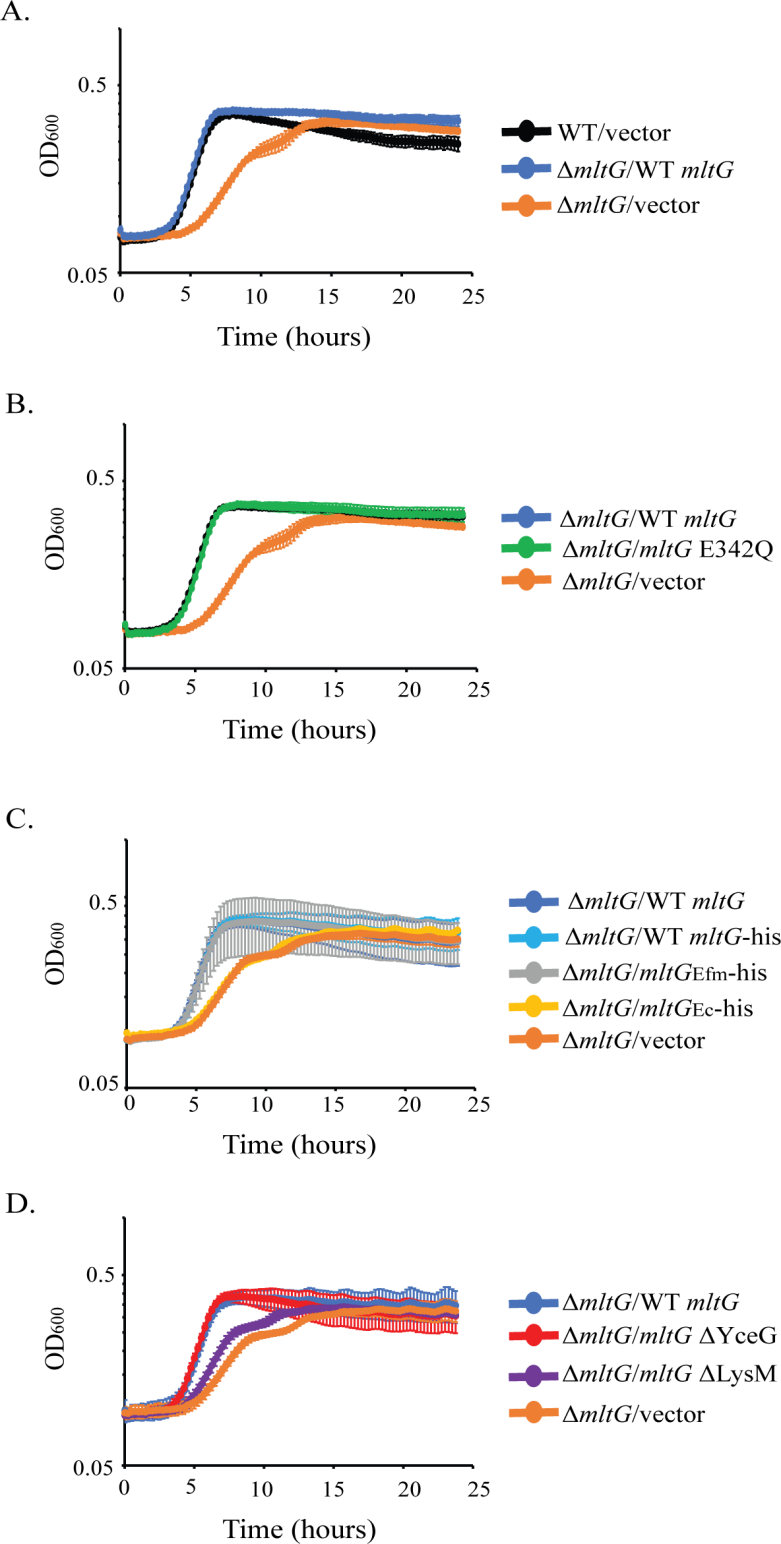
MltG deletion results in a growth defect. Bacterial growth (OD_600_) was monitored over the course of 24 hours. Data represent the mean ± standard deviation of three biological replicates (two replicates for WT/vector). Error bars are too small to be seen in some cases. Strains were (**A**) WT/vector = OG1(pJRG9); Δ*mltG/*WT *mltG =* JL650(pAAU12); Δ*mltG/*vector = JL650(pJRG9); (**B**) Δ*mltG/*WT *mltG =* JL650(pAAU12); Δ*mltG*/*mltG* E342Q *=* JL650(pAAK15); Δ*mltG/*vector = JL650(pJRG9); (**C**) Δ*mltG/*WT *mltG =* JL650(pAAU12); Δ*mltG/*WT *mltG-his =* JL650(pAAK33); Δ*mltG/mltG_Efm_-his =* JL650(pAAK76); Δ*mltG/mltG_Ec_-his =* JL650(pAAK75); Δ*mltG/*vector = JL650(pJRG9); and (**D**) Δ*mltG/*WT *mltG =* JL650(pAAU12); Δ*mltG/mltG* ΔYceG = JL650(pAAK65); Δ*mltG/mltG* ΔLysM = JL650(pAAK67); Δ*mltG/*vector = JL650(pJRG9).

To test whether MltG plays a role in PG homeostasis in growing *E. faecalis*, we constructed an in-frame deletion of *mltG* from the chromosome, using previously described methods (39, 40). We observed that the Δ*mltG* mutant exhibited a growth defect, in that its exponential growth was delayed and occurred at a slower rate compared to that of the wild type (Fig. 2A). This growth defect could be complemented by ectopic expression of WT MltG, which was expressed to similar levels as MltG expressed from the chromosome (Fig. 2A; Fig. S2A), confirming that the loss of MltG was responsible for the growth defect. To determine whether the catalytic activity of MltG was important for growth, we also analyzed the Δ*mltG* mutant with ectopic expression of MltG E342Q (Fig. S2B). The catalytically inactive MltG E342Q mutant supported wild-type growth, suggesting that another function of MltG (independent of its catalytic activity) is necessary to support wild-type growth (Fig. 2B).

*Enterococcus faecium* encodes a MltG homolog, previously unstudied, that is ~52% identical to *E. faecalis* MltG at the amino acid level. To determine whether *E. faecium* MltG (MltG_Efm_) is a functional homolog of *E. faecalis* MltG, we expressed MltG_Efm_ in *trans* within the *E. faecalis* Δ*mltG* strain (Fig. S3B). We found that MltG_Efm_ complemented the growth defect of the *E. faecalis* Δ*mltG* mutant, indicating that MltG_Efm_ is a functional homolog of *E. faecalis* MltG (Fig. 2C).

### Deletion of *mltG* is detrimental to cell wall integrity

**Fig 3:**
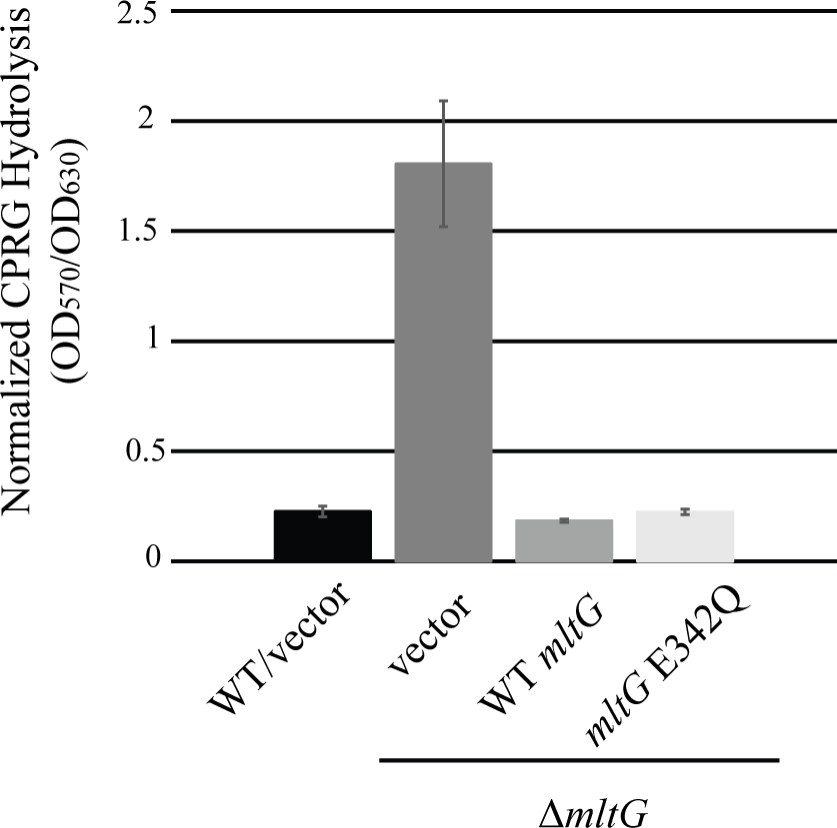
MltG deletion leads to a loss of cell wall integrity. Strains constitutively producing β-galactosidase were grown for 24 hours in MHB in the presence of CPRG (β-galactosidase substrate). CPRG cleavage (OD_570_) normalized to bacterial growth (OD_630_) is presented. *n* = 3 and error bars represent ±s.d. Strains were WT/vector = OG1(pJRG9); Δ*mltG*/vector = JL650(pJRG9); Δ*mltG*/WT *mltG* = JL650(pAAU12); Δ*mltG*/*mltG* E342Q = JL650(pAAK15).

Due to the proposed role of MltG in PG homeostasis and the growth defect of the Δ*mltG* strain, we hypothesized that MltG was important for cell wall integrity in *E. faecalis*. To test this, we used a previously described assay to measure LacZ-mediated hydrolysis of chlorophenol red galactopyranoside (CPRG). CPRG is normally excluded from wild-type *E. faecalis* cells and therefore does not get significantly hydrolyzed by cytoplasmic LacZ. However, mutants in which the integrity of the cell wall is compromised can enable access of CPRG to LacZ and subsequent hydrolysis to yield a red product. As expected, wild-type cells exhibited limited CPRG cleavage (Fig. 3). By contrast, the Δ*mltG* mutant exhibited a substantial increase in CPRG hydrolysis, which could be complemented by expression of both WT MltG and MltG E342Q (Fig. 3), confirming that the absence of MltG was responsible. Together, these results indicate that the cell wall integrity of the Δ*mltG* mutant is compromised.

**Fig 4:**
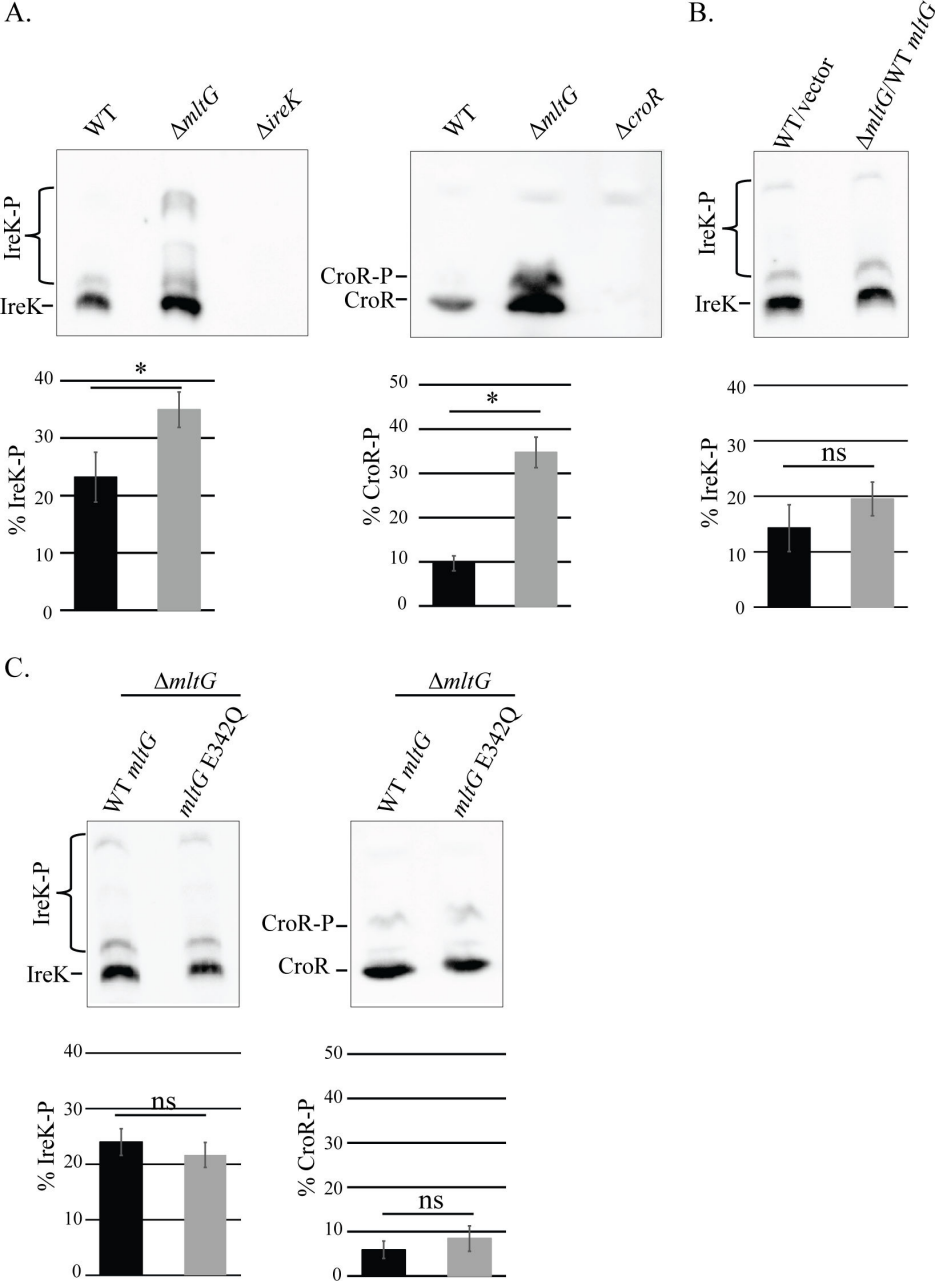
MltG deletion triggers cell wall stress signaling. For each pairwise comparison, whole-cell lysates were subjected to phos-tag SDS-PAGE and then immunoblotting for IreK or CroR as indicated. The upper band(s) represent phosphorylated proteoform(s). Intensities from the phosphorylated and unphosphorylated signals were used to calculate % phosphorylation of IreK or CroR, presented as the average % phosphorylation in bar graphs. (**A**) Comparison of WT vs Δ*mltG* reveals that MltG deletion triggers phosphorylation of IreK and CroR. Comparison of (**B**) WT/vector vs Δ*mltG/*WT *mltG* or (**C**) Δ*mltG* expressing either WT *mltG* or Δ*mltG* /*mltG* E342Q reveals complementation of the Δ*mltG* mutation. *n* = 3 and error bars represent ±s.d. *P < .05; ns = not significant. Student’s t-test (heteroscedastic, two-tailed). Strains were WT = OG1; Δ*mltG* = JL650; Δ*ireK =* JL206; Δ*croR* = SB23; WT/vector = OG1(pJRG9); Δ*mltG/*WT *mltG* = JL650(pAAU12); Δ*mltG*/*mltG* E342Q *=* JL650(pAAK15).

Defects in cell wall integrity could potentially be detected by the cells using cell-wall-stress-responsive signal transduction systems. To determine whether the impaired cell wall integrity of the Δ*mltG* mutant triggered cell wall stress signaling systems, we assessed activation of cell wall stress signaling by monitoring phosphorylation of IreK and CroR (which are both known to be phosphorylated in response to cell wall stress (26, 29, 31)). To do so, we used phos-tag SDS-PAGE, which enables the separation of phosphorylated proteoforms by impeding their migration through the SDS-PAGE gel (29–31, 41). In exponentially growing cells that had not been exposed to any exogenous cell wall stressors, we found that both IreK and CroR phosphorylation were elevated in the Δ*mltG* mutant as indicated by the appearance of more slowly migrating phosphorylated proteoforms known to be induced by cell wall stress (Fig. 4A), which could be complemented by ectopic expression of WT MltG (Fig. 4B) and the catalytically inactive MltG E342Q (Fig. 4C). Together, these results indicate that the loss of MltG triggers both the IreK and CroR cell wall stress signaling systems, consistent with a defect in cell wall integrity in the Δ*mltG* mutant, and that some function of MltG other than its enzymatic activity is required for maintenance of *E. faecalis* cell wall integrity.

**Fig 5:**
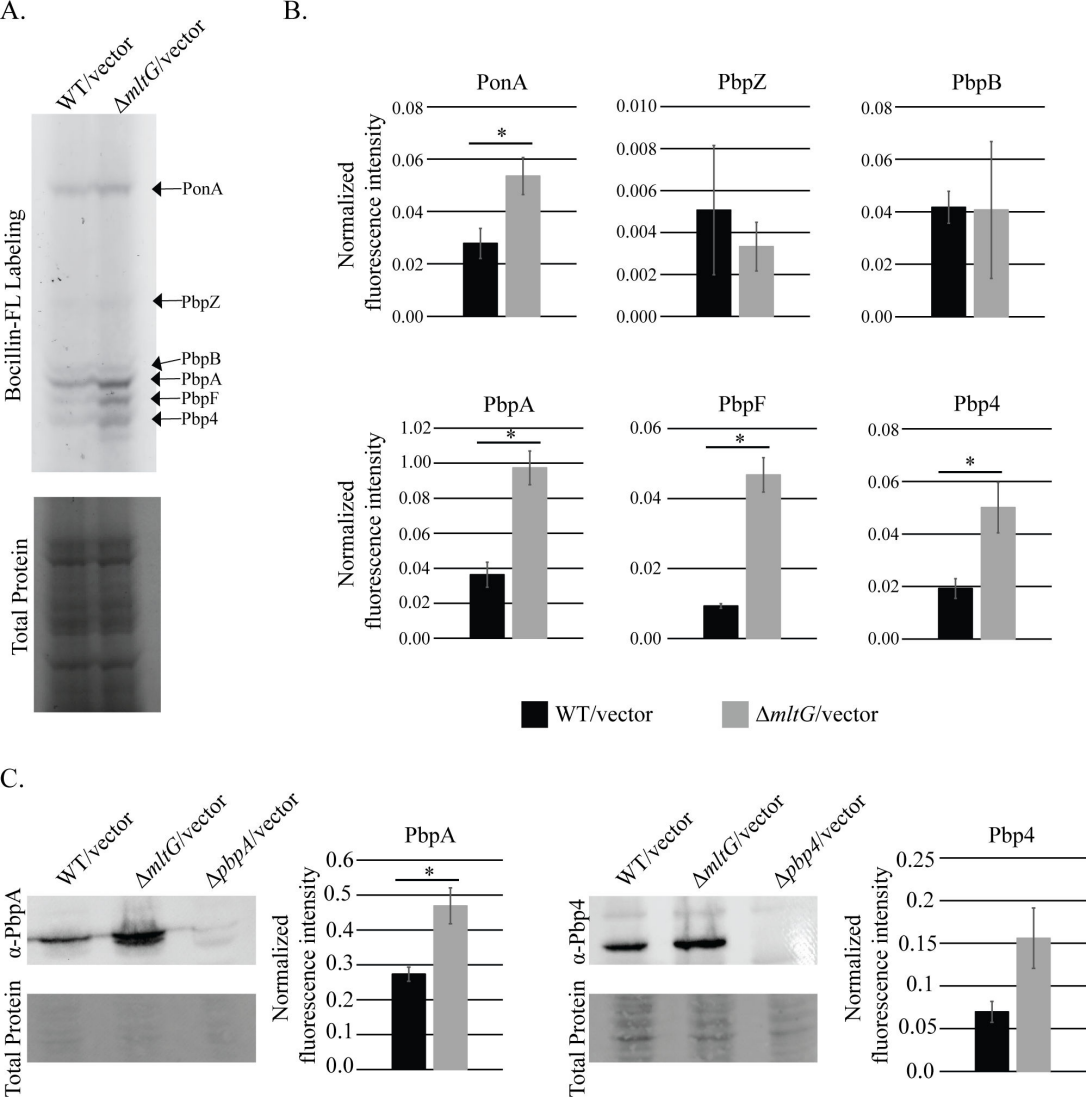
MltG deletion increases PBP expression. Strains grown to exponential phase were exposed to Bocillin-FL, which acylates active PBPs. (**A**) Labeled cells were then lysed and subjected to SDS-PAGE. The image is representative of three biological replicates. (**B**) The intensity of Bocillin-FL labeling for each PBP was normalized to total protein staining (GelCode Blue). Graphs represent the average of three biological replicates. (**C**) Cell lysates were subjected to SDS-PAGE supplemented with TCE for total protein detection and immunoblot with antiserum for PbpA or Pbp4. Graphs represent the average of three biological replicates. Error bars represent ±s.d. *P< .05; Student’s t-test (heteroschidastic, two-tailed). Strains were WT/vector = OG1(pJRG9); Δ*mltG/*vector *=* JL650(pJRG9); Δ*pbpA/*vector = JL632(pJRG9); Δ*pbp4/*vector = JL339(pJRG9).

Previous studies reported that MltG homologs can interact with PG synthases and described an antagonistic relationship between MltG homologs and PBPs (33, 35–38). To test whether PBP activity was altered upon deletion of *mltG* in *E. faecalis,* we used Bocillin-FL to label active *E. faecalis* PBPs *in vivo* (21). Bocillin-FL is a fluorescent activity-based probe that acylates the active site of catalytically active PBPs. Bocillin-FL labeling of exponentially growing cells revealed that the Δ*mltG* strain exhibited increased Bocillin-FL labeling of multiple PBPs, including PbpA and Pbp4 (Fig. 5A and B), which are known to be transcriptionally upregulated by the CroR signaling system (32). It is unknown whether the transpeptidase activity of PbpA or Pbp4 is regulated in cells; therefore, formally the increase in Bocillin-FL labeling could be due to either changes in the extent of PBP activity or to changes in the abundance of the PBPs. To determine whether the increase in Bocillin-FL labeling of PbpA and Pbp4 was due to an increase in PBP activity or PBP expression, the lysates were probed by immunoblot. PbpA and Pbp4 (although Pbp4 was not statistically significant) exhibited an increase in protein abundance upon deletion of *mltG* (Fig. 5C), suggesting that the observed increase in Bocillin-FL labeling is likely due to upregulation of PBP expression in the Δ*mltG* mutant, mediated by activation of CroR signaling (Fig. 4A). However, we cannot rule out a potential change in PBP activity as well. Nevertheless, these data indicate that activation of the CroR cell-wall-stress signaling system results in enhancement of expression for PbpA and Pbp4, which is expected during the response to cell wall stress.

**Fig 6:**
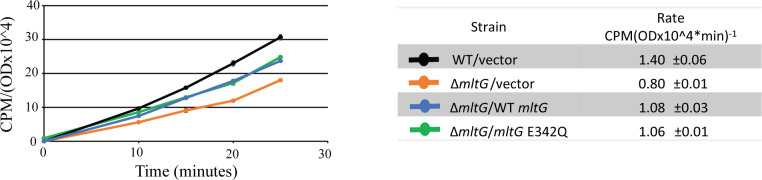
MltG deletion reduces the rate of PG synthesis. [^14^C] GlcNAc incorporation into PG during pulse-labeling, normalized to cell density. Label incorporation rates presented in the table were determined from data points between 10 and 25 minutes. Data represent ±s.d. for two biological replicates. Strains were WT/vector = OG1(pJRG9); Δ*mltG/*vector *=* JL650(pJRG9); Δ*mltG/*WT *mltG* = JL650(pAAU12); Δ*mltG*/*mltG* E342Q *=* JL650(pAAK15).

To explicitly test whether the loss of MltG affected PG synthesis, we assayed the rate of cell wall synthesis by measuring incorporation of [^14^C]GlcNAc into SDS-insoluble PG during exponential growth (21, 23, 24). The Δ*mltG* strain exhibited a decreased rate of PG synthesis (Fig. 6), which likely contributes to the defect in cell wall integrity described above. Complementation with ectopic MltG partially restored PG synthesis of the mutant. It is noteworthy that the MltG E342Q mutant also restored PG synthesis to the same extent as wild-type MltG (Fig. 6), indicating that a function of MltG other than its enzymatic activity is required to promote PG synthesis in *E. faecalis*.

### Deletion of *mltG* enhances ceftriaxone resistance through activation of IreK and CroR

We previously showed that activation of the IreK and CroR cell wall stress signaling systems drive enhanced resistance to cephalosporins (26, 29, 31). To test the hypothesis that the Δ*mltG* mutant might exhibit elevated cephalosporin resistance as a consequence of IreK or CroR activation, we determined minimum inhibitory concentrations (MICs) for ceftriaxone, a representative cephalosporin. We found that the Δ*mltG* strain exhibited a hyper-resistant phenotype toward ceftriaxone, which was complemented by ectopic expression of WT MltG (Table 1). Thus, MltG is not itself strictly required for cephalosporin resistance, and the hyper-resistant phenotype is consistent with enhanced activation of IreK and CroR signaling. To specifically determine whether the catalytic activity of MltG influenced ceftriaxone resistance, we complemented the Δ*mltG* strain with catalytically inactive MltG E342Q. Surprisingly, although MltG E342Q does not trigger cell wall stress signaling (Fig. 4C), MltG E342Q also drove hyper-resistance toward ceftriaxone (Table 1), indicating that MltG enzymatic activity can modulate cephalosporin resistance.

**TABLE 1:** MltG deletion and inhibition of catalytic activity lead to cephalosporin hyper-resistance

Strain^*a*^	Ceftriaxone MIC (µg/mL)^*b*^
WT/vector	64
Δ*mltG*/vector	512
Δ*mltG*/WT *mltG*	64
Δ*mltG*/*mltG* E342Q	512

^*a*^ Strains were WT/vector = OG1(pJRG9); Δ*mltG*/vector = JL650(pJRG9); Δ*mltG/*WT *mltG* = JL650(pAAU12); Δ*mltG/mltG* E342Q = JL650(pAAK15).

^*b*^ The median MIC of ceftriaxone was determined from at least three biological replicates.

To determine whether the hyper-resistance exhibited by the Δ*mltG* strain was dependent on activation of cell wall stress signaling systems, we pharmacologically or genetically impaired each system. First, to test whether IreK signaling is essential to mediate ceftriaxone hyper-resistance upon deletion of *mltG*, we performed MICs in the presence of staurosporine, which inhibits IreK kinase activity (28). Staurosporine led to a reduction in ceftriaxone resistance of the wild type, as expected upon inhibition of IreK. Importantly, staurosporine also led to a reduction in ceftriaxone resistance of the Δ*mltG* mutant, even more substantially than in wild-type cells (Table 2). We conclude that activation of IreK-mediated signaling is required for the ceftriaxone hyper-resistance observed with the Δ*mltG* mutant.

**TABLE 2:** IreK is required for hyper-resistance of the Δ*mltG* mutant

	Ceftriaxone MIC (µg/mL)^*a*^	
Strain^*b*^	DMSO	Staurosporine
WT/vector	64	8
Δ*mltG*/vector	1,024	<4
Δ*mltG*/WT *mltG*	128	8

^*a*^ The median MIC of ceftriaxone was determined from at least three biological replicates in the presence of vehicle control DMSO or 10 µM Staurosporine.

^*b*^ Strains were WT/vector = OG1(pJRG9); Δ*mltG*/vector = JL650(pJRG9); Δ*mltG/*WT *mltG* = JL650(pAAU12).

To test whether the activation of CroR was also required for hyper-resistance, we attempted to construct a Δ*mltG* Δ*croR* double mutant; however, we were unable to successfully obtain such a double mutant, suggesting that the double deletion mutant is not viable. Therefore, we constructed a *mltG*-depletion Δ*croR* double mutant (dep-*mltG* Δ*croR*) in which the chromosomal copy of *mltG* was deleted from the Δ*croR* mutant in the presence of an inducible copy of *mltG*. Analysis of ceftriaxone resistance (Table 3) revealed that the hyper-resistance normally observed upon depletion of MltG (0 mM NaNO_3_ inducer) was lost in the absence of CroR, indicating that CroR is required for the hyper-resistance exhibited by the Δ*mltG* strain. We previously showed that CroR-mediated upregulation of Pbp4 is required for CroR-driven cephalosporin resistance (32). To test the importance of CroR-mediated Pbp4 upregulation in hyper-resistance of the Δ*mltG* mutant, we constructed a Δ*mltG* Δ*pbp4* double mutant. Analysis of ceftriaxone resistance revealed that deletion of *pbp4* eliminated hyper-resistance upon deletion of *mltG* and that expression of Pbp4 from an inducible vector restored ceftriaxone resistance (Table 3). We therefore conclude that the Δ*mltG* mutant requires activation of signaling through IreK and CroR, including (but not limited to) CroR-mediated upregulation of Pbp4 to drive hyper-resistance towards cephalosporins.

**TABLE 3:** Dependence on CroR signaling to mediate hyper-resistance

	Ceftriaxone MIC (µg/mL)^*a*^	
Strain^*b*^	0 mM Inducer^*c*^	25 mM Inducer
WT/vector	128	128
Δ*mltG*/vector	>256	>256
Δ*croR*/vector	8	8
dep-*mltG* Δ*croR /*WT *mltG*	2	8
Δ*pbp4*/vector	<1	<1
Δ*mltG* Δ*pbp4*/vector	<1	<1
Δ*mltG* Δ*pbp4*/WT *pbp4*	<1	128

^*a*^ The median MIC of ceftriaxone determined from at least three biological replicates (two for Δ*pbp4*/vector).

^*b*^ Strains were WT/vector = OG1(pJLL286); Δ*mltG*/vector = JL650(pJLL286); Δ*croR*/vector = SB23(pJLL286); dep-*mltG* Δ*croR /*WT *mltG* = AK6(pNPC1); Δ*pbp4*/vector = JL339(pJLL286); Δ*mltG* Δ*pbp4*/vector = JL704(pJLL286); Δ*mltG* Δ*pbp4*/WT *pbp4* = JL704(pJLL371).

^*c*^ NaNO_3_ used to induce protein expression from pJLL286-derived plasmids.

### The LysM domain is critical for MltG function

The observation that MltG E342Q leads to abnormally elevated ceftriaxone resistance (Table 1) indicates that the E342Q variant must be enzymatically inactive when expressed in *E. faecalis* cells, just as it is inactive *in vitro* (Fig. 1). However, MltG E342Q rescues multiple phenotypic defects of the Δ*mltG* mutant, including the growth defect (Fig. 2), loss of cell wall integrity (Fig. 3), activation of cell wall stress signaling systems (Fig. 4), and PG synthesis (Fig. 6), leading us to hypothesize that a non-enzymatic function of MltG is critical. Previous studies of MltG homologs have shown that the LysM domain, a putative PG-binding domain, is essential to support growth (38), suggesting that the LysM domain of *E. faecalis* MltG might provide the critical non-enzymatic function. To test this, we constructed and ectopically expressed MltG variants specifically lacking either the LysM domain (ΔLysM) or the enzymatic YceG domain (ΔYceG). To facilitate immunoblot analysis, we included a C-terminal His_6_ tag on all MltG variants. Immunoblotting revealed that both MltG domain deletion variants were stably expressed in *E. faecalis* at their respective expected molecular weight (Fig. S3C). His-tagged wild-type MltG complemented the growth of the Δ*mltG* mutant, indicating that the His tag did not impair MltG function. The MltG ΔYceG variant complemented the growth defect of the Δ*mltG* mutant and drove hyper-resistance toward ceftriaxone (Fig. 2D; Fig. S4; Table S1), phenocopying the E342Q variant. Thus, we conclude that not only the enzymatic activity but the entire YceG domain itself is dispensable for MltG to support growth (and presumably cell wall integrity). By contrast, we found that the MltG ΔLysM variant could not complement the growth of the Δ*mltG* mutant (Fig. 2D; Fig. S4), suggesting the LysM domain is specifically required for this function. As an additional test of this, we expressed full-length *E. coli* MltG-His_6_, whose LysM domain is substantially different from the LysM domain of *E. faecalis* MltG at the amino acid sequence level (~25% identical), in the *E. faecalis* Δ*mltG* mutant. Although *E. coli* MltG was expressed in *E. faecalis* (Fig. S3B), it could not rescue the growth of the Δ*mltG* mutant (Fig. 2). Together, these results support the hypothesis that the LysM domain of MltG is specifically important to support growth and cell wall homeostasis.

## DISCUSSION

The lytic transglycosylase, MltG, has been characterized in several bacterial species where it has been shown to cleave nascent strands of PG and is proposed to act as a terminase during synthesis of the glycan backbone of PG, thereby priming the resulting PG strands for transpeptidation and integration into the mature cell wall (33–38). However, the function of enterococcal MltG has not been investigated previously. Given the proposed function of MltG in PG homeostasis, we hypothesized that MltG might impact enterococcal resistance toward cell wall active antibiotics such as cephalosporins, for which resistance relies on multiple factors involved in PG synthesis (21–25). Here, we demonstrate that enterococcal MltG cleaves nascent PG. An *E. faecalis* mutant lacking MltG exhibits several related phenotypes in the absence of exogenous stress: a marked growth defect, a loss of cell wall integrity, a reduction in PG synthesis, and activation of cell wall stress signal transduction systems that drive elevated cephalosporin resistance. Together, these results are consistent with the model that MltG promotes proper cell wall homeostasis in *E. faecalis*. However, our results also reveal that the enzymatic activity of MltG is not necessary for it to perform this function—instead, the MltG LysM domain plays the critical role.

Unexpectedly, we found that deletion of *mltG* drives hyper-resistance toward ceftriaxone. This contrasts with previous studies conducted in *Neisseria gonorrhoeae* and *Pseudomonas aeruginosa,* which described a loss of resistance toward cell wall active antibiotics, including ceftriaxone and other beta-lactam antibiotics, upon deletion of *mltG* (36, 42–44). For *P. aeruginosa*, loss of resistance toward beta-lactams upon deletion of *mltG* is due to a lack of *ampC* expression in the absence of the MltG cleavage product, 1,6-anhydrous MurNAc, which normally de-represses *ampC* transcription (42). Perturbations of PG recycling are also implicated in the *N. gonorrhoeae* MltG deletion strain, which may be acting by a similar mechanism (36). *E. faecalis* does not possess a known PG recycling system, although it has been hypothesized that some cell wall stress signaling systems might sense extracellular PG fragments as a method of cell wall stress detection (45–47).

**Fig 7:**
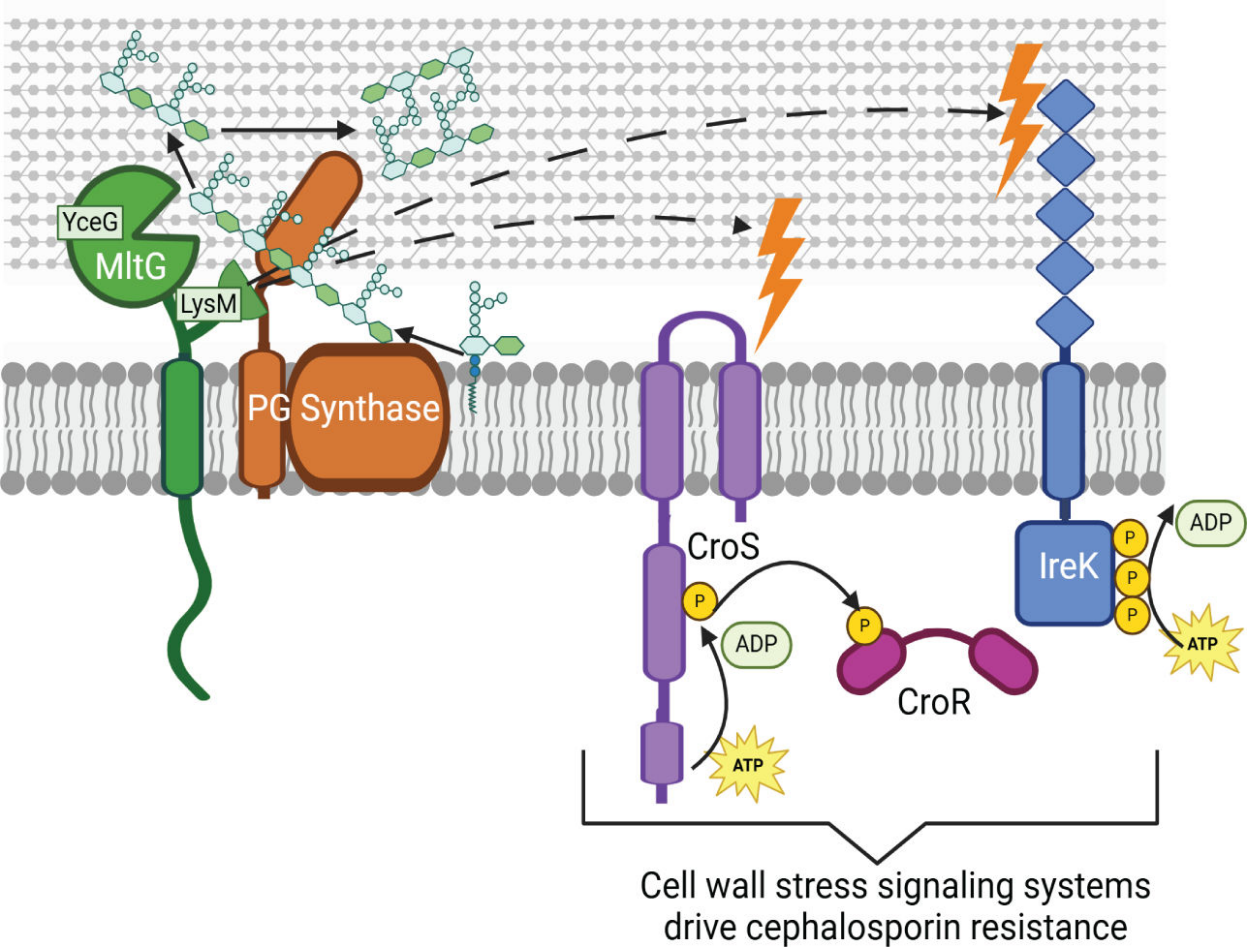
Summary model. Our data support a model in which *E. faecalis* MltG cleaves nascent strands of PG through the action of its C-terminal YceG domain, presumably to enable proper incorporation of nascent PG strands into mature PG. In addition, the membrane-proximal LysM domain of MltG appears essential for the ability of MltG to support normal growth and cell wall integrity, because loss of the LysM domain (but not the catalytic YceG domain) results in activation of the CroRS and IreK cell wall stress signaling systems to drive hyper-resistance toward cephalosporins. Created in BioRender. Uitenbroek, A. (2025) https://BioRender.com/2l14ekc

Here, we demonstrate that the hyper-resistance exhibited by the Δ*mltG* mutant is dependent on activation of cell wall stress signaling systems IreK and CroRS (Fig. 7). Our results indicate that IreK and CroRS are activated in the Δ*mltG* mutant (Fig. 4), likely in response to the reduction in cell wall integrity (Fig. 3), which is presumably a consequence of impaired PG synthesis (Fig. 6). We found that upon pharmacologic inhibition of IreK or chromosomal deletion of *croR*, cells deficient in MltG no longer exhibited hyper-resistance toward ceftriaxone. In addition, Pbp4—a known CroR regulon member and determinant of ceftriaxone resistance—was shown to be upregulated in the Δ*mltG* strain. Upon deletion of *pbp4*, the Δ*mltG* mutant no longer exhibited hyper-resistance toward ceftriaxone, further supporting that CroR gene regulation is essential to promote resistance of the Δ*mltG* strain. We note that upon inhibition of cell wall stress signaling systems, deletion of *mltG* compromised ceftriaxone resistance even further (Tables 2 and 3). This suggests that MltG actually helps promote ceftriaxone resistance (although perhaps only modestly), and that this effect is masked in an otherwise wild-type background due to the activation of cell wall stress signaling systems.

We found that inhibition of MltG catalytic activity via mutation of its conserved catalytic residue (E342Q) or truncation of the catalytic YceG domain drove hyper-resistance toward ceftriaxone to the same degree as the Δ*mltG* mutant. However, expression of MltG E342Q did not result in other Δ*mltG* mutant-like phenotypes, having no impact on growth, rate of PG synthesis, cell wall integrity, or activation of cell wall stress signaling systems. This suggests that impairment of MltG catalytic activity drives cephalosporin resistance by a unique mechanism. Although the underlying mechanism remains unknown, we speculate that inhibition of MltG catalytic activity may increase the transpeptidase activities of PBPs. Genetic studies have revealed an antagonistic relationship between MltG homologs and PBPs, and Sassine et al. demonstrated that *E. coli* and *Bacillus subtilis* MltG homologs reduce the transpeptidase activity of PBPs *in vitro* (33, 35, 37, 38). An increase in the activity of “low-reactivity” PBPs in particular, such as Pbp4 and PbpA, that are capable of catalyzing PG transpeptidation despite the presence of cephalosporins in the environment, might be expected to confer additional resistance toward cephalosporins. Future studies will focus on understanding the molecular mechanism by which impairment of MltG catalytic activity drives cephalosporin resistance.

Our results indicate that *E. faecalis* MltG does more than just catalyze the cleavage of nascent PG. A MltG mutant lacking its catalytic YceG domain supported wild-type growth (as also observed with the E342Q catalytically inactive mutant). Specific deletion of the putative PG-binding LysM domain resulted in impaired growth, suggesting that the LysM domain function is essential to normal *E. faecalis* growth. Moreover, heterologous expression of full-length wild-type *E. coli* MltG did not rescue the growth of the *E. faecalis* Δ*mltG* mutant, consistent with the hypothesis that the LysM domain of *E. faecalis* MltG performs a key function that the divergent *E. coli* LysM domain cannot provide. Overall, our results are consistent with the findings of Bohrhunter et al., in which the LysM domain of the *E. coli* MltG homolog was necessary for functional complementation of a salt-sensitive *mltG* deletion strain (38). Further studies are needed to investigate the functional role of the putative PG-binding LysM domain of *E. faecalis* MltG.

## MATERIALS AND METHODS

### Bacterial strains and growth conditions

Bacterial strains and plasmids used in this study are listed in Table S2. *E. coli* strains were grown in Lysogeny broth (LB; Lennox formulation) or agar (Difco). *E. faecalis* was grown in Mueller-Hinton broth (MHB) or agar (Difco) at 30°C unless otherwise indicated. All cultures were grown aerobically with shaking (225 rpm). As appropriate, 10 µg/mL chloramphenicol, 10 µg/mL (for *E. faecalis*), 100 µg/mL (for *E. coli*) erythromycin, or 50 µg/mL kanamycin was used for maintenance of plasmids.

### Genetic manipulation of enterococci

Mutant strains were constructed using the temperature-sensitive, counter-selectable allelic exchange plasmid pJH086 as previously described (40). In addition to p-Cl-Phe, the counter-selection plates contained 20% sucrose. Regions of genomic DNA flanking the gene to be deleted were amplified by PCR and introduced to pJH086 by isothermal assembly (48). Deletion alleles were in-frame and retained a variable number of codons at the beginning and end of each gene, to avoid perturbing the expression of adjacent genes. Table S2 contains the specific details of deletion alleles. All mutant or complemented strains were constructed independently at least twice and analyzed to ensure that their phenotypes were concordant. Construction of the dep-*mltG* Δ*croR* strain was carried out with cells carrying an inducible copy of *mltG* expressed from pJLL286 in the presence of erythromycin. In these cases, 10 µg/mL erythromycin and 25 mM NaNO3 were included in the counterselection plates and the growth media for all subsequent propagation, except as noted for specific experiments.

### Construction of plasmids

All recombinant plasmids were constructed using Gibson Assembly (48). The full insert of all constructs was sequenced to confirm the absence of mutations. MltG (wild type and mutants) was ectopically expressed in *E. faecalis* OG1 background using the enterococcal expression plasmid pJRG9 under the control of the constitutive P23s promoter and an artificial ribosome-binding site (TGGAGGAACTCAAT). For purification from *E. coli*, MltG (wild type and mutants) was expressed from the isopropyl βeta-D-1-thiogalactopyranoside (IPTG)-inducible expression vector pET28a-His-*smt3*, which encodes a His_6_-SUMO fusion.

### Lipid II extraction

Lipid II from *E. faecalis* cells was obtained through sequential extractions using chloroform:methanol and pyridinium acetate:n-butanol as described by Welsh et al. (49). Extracted Lipid II was quantified using fluorescamine (a compound which fluoresces upon interaction with primary amines present in Lipid II) normalized to a standard curve as described by Sardis et al. (50).

### Pbp1A purification

The extracellular domain of recombinant Pbp1A (*Streptococcus pneumoniae* SPD_0336 V36-P719) was expressed and purified from BL21(DE3) cells. Stationary-phase cultures were diluted 1:200 into 1 L of LB media supplemented with 50 µg/mL kanamycin for plasmid maintenance and grown with shaking at 37°C until OD_600_ reached 0.5–0.6 and then shifted to 16°C. Protein expression was then induced by the addition of 0.5 mM IPTG and incubated at 16°C for 22 hours with shaking. Cells were collected by centrifugation (4,000 rpm 15 minutes at 4°C) and resuspended in 25 mL Buffer A (50 mM HEPES pH 7.5, 500 mM NaCl, 0.5% CHAPS). Cells were then lysed by the French press and centrifuged (48,000 × *g* for 50 minutes at 4°C) to remove insoluble cell debris. The supernatant was then incubated with 1 mL of Ni-charged resin (BioRad) equilibrated with 10 column volumes of Buffer A, and imidazole was added to a final concentration of 10 mM. The resin was then washed with 10 column volumes of Buffer A + 20 mM imidazole and 10 column volumes of Buffer A + 40 mM imidazole. Protein was eluted in 2 mL fractions with Buffer A + 250 mM imidazole. Eluted protein was concentrated and resuspended in Buffer A to remove imidazole. Protein purity and concentration were evaluated by subjecting the purified protein to SDS-PAGE and GelCode Blue (Thermo Scientific) staining. Protein concentration was determined by creating a standard curve of a series of known BSA concentrations using the band intensities in ImageLab (BioRad) and extrapolating the desired protein concentration from a dilution series of the protein on the same gel. Aliquots of purified protein were stored at −80°C.

### MltG purification

Recombinant full-length protein, WT, and E342Q His_6_-SUMO-MltG were expressed and purified from C43(DE3) cells. For each protein, stationary-phase cultures were diluted 1:200 in two 500 mL cultures of LB media with 50 µg/mL kanamycin for plasmid maintenance and grown with shaking at 37°C until OD_600_ reached 0.5–0.6 and then shifted to 16°C. Protein expression was then induced by the addition of 0.4 mM IPTG and incubated at 16°C for 18 hours with shaking. The two cultures were pooled, and cells were collected by centrifugation (4,500 rpm for 15 minutes at 4°C), stored at −80°C, thawed on ice, and resuspended in 40 mL Lysis buffer (50 mM Tris-HCl [pH 7.4], 150 mM NaCl, 10% [vol/vol] glycerol), with 1:100 HALT protease inhibitor cocktail (Thermo Fisher Scientific) and 1:100 DNAse. Cells were then lysed by French press. The membrane fraction was collected by ultracentrifugation (46,000 × *g* for 2 hours at 4°C). The membrane pellet was stored at −80°C, thawed on ice, and resuspended in 20 mL Solubilization Buffer (1% [wt/vol] n-Dodecyl β-D-maltoside [DDM], 20 mM Tris-HCl [pH 7.4], 500 mM NaCl, 10% [vol/vol] glycerol) with 1:100 HALT protease inhibitor cocktail. The resulting mixture was passed through 21- and 18-gauge needles and stirred for 1 hour at 4°C. Insolubilized protein was removed by ultracentrifugation (46,000 × *g* for 1 hour at 4°C). The supernatant containing solubilized membrane protein was passed through a 0.22 µm filter to remove cell debris. The filtered supernatant was incubated with 3 mL of Ni-charged resin (BioRad) equilibrated with 10 column volumes of Solubilization Buffer. The resin was then washed with 20 column volumes of Wash Buffer (20 mM Tris-HCl [pH 7.4], 500 mM NaCl, 10% [vol/vol] glycerol, 0.01% [wt/vol] DDM, 20 mM imidazole [pH 7.4]). Protein was eluted in 2 mL fractions with Elution Buffer (500 mM imidazole [pH 7.4], 20 mM Tris-HCl [pH 7.4], 500 mM NaCl, [vol/vol] 10% glycerol, 0.01% [wt/vol] DDM). Imidazole was removed with 1 L Dialysis Buffer (20 mM Tris-HCl [pH 7.4], 500 mM NaCl, 0.01% [wt/vol] DDM) for 1 hour at 4°C. The His_6_-SUMO-tag was removed from the desired protein by adding His-GB1-Ulp1 protease and 0.5 mM DTT to the sample before dialysis with a fresh 1 L Dialysis Buffer at 4°C overnight. After dialysis and Ulp1 cleavage, proteins were incubated with a 2 mL Ni-charged resin (BioRad) equilibrated with Dialysis Buffer for 30 minutes at 4°C with rocking. After the incubation, the flow-through containing the untagged desired protein was collected. Protein purity and concentration were evaluated by subjecting the purified protein to SDS-PAGE and GelCode Blue (Thermo Scientific) staining. Protein concentration was determined by creating a standard curve of a series of known BSA concentrations using the band intensities in ImageLab (BioRad) and extrapolating the desired protein concentration from a dilution series of the protein on the same gel. Aliquots of purified protein were stored at −80°C. Melt curve of protein was assessed by Tycho NT.6 (NanoTemper) before and after storage at −80°C.

### PbpX purification

His_6_-PbpX T36-P429 was expressed and purified from Nico21 (DE3) cells as previously described by Nelson et al. (25). Stationary-phase cultures were diluted 1:200 in 1L of LB media with 50 µg/mL kanamycin for plasmid maintenance and grown with shaking at 37°C until OD_600_ reached 0.5–0.6 and then shifted to 20°C. Protein expression was induced with 1 mM IPTG, and the culture was incubated at 20°C overnight with shaking. Cells were collected by centrifugation (4,500 rpm for 15 minutes at 4°C), resuspended in 40 mL binding buffer (50 mM Tris [pH 8.0], 300 mM NaCl, 5 mM imidazole [pH 8.0]), and lysed by a French press. The membrane fraction was collected by ultracentrifugation (48,000 × *g* for 1 hour at 4°C). The supernatant containing cytosolic protein was passed through a 0.22 µm filter to remove cell debris. Filtered supernatant was applied to a 2 mL bed volume of Ni-charged resin (BioRad) equilibrated in 10-column volumes of binding buffer. The resin was then washed with 20 column volumes of wash buffer (50 mM Tris pH = 8.0, 300 mM NaCl, 20 mM Imidazole), and the protein was eluted in 2 mL fractions with elution buffer (50 mM Tris [pH 8.0], 300 mM NaCl, 500 mM Imidazole [pH 8.0]). Imidazole was removed with 1 L dialysis buffer (50 mM Tris [pH 8.0], 150 mM NaCl, 10% [vol/vol] glycerol) for 1 hour at 4°C and a fresh 1 L dialysis buffer overnight at 4°C. Protein purity was evaluated by subjecting the purified protein to SDS-PAGE and GelCode Blue staining. Protein concentration was determined using the Bradford Assay.

### Preparation of BDL

Biotinylated-D-lysine was prepared from Fmoc-D-Lys(biotynyl)-OH by the addition of 20% piperidine/DMF and toluene, concentration to remove solvent, and resuspension in H_2_O, followed by filtration to remove precipitant as previously described by Qiao et al. (51)

### *In vitro* PG cleavage assay

The *in vitro* PG cleavage assay was performed as previously described (25, 34, 52), with modifications. To synthesize PG strands, 2 µM purified Pbp1A was incubated with 200 µM purified lipid II in reaction buffer (50 mM HEPES pH 7.5, 2.5 mM MgCl_2_, 20 mM CaCl_2_, 30% DMSO, supplemented with 7.3 mM Tris pH 7.4, 182.5 mM NaCl, 0.002% DDM). Reactions were run overnight at 25°C, quenched by incubation at 98°C for 5 minutes, and cooled to room temperature. To cleave (or not) PG strands, purified WT MltG or MltG E342Q (1.5 µM) was then added to the reaction, which was brought to 20 µL with MltG dialysis buffer for a final reaction composition of 50 mM HEPES pH 7.5, 2.5 mM MgCl2, 20 mM CaCl2, 15% DMSO, 12.65 mM Tris pH 7.4, 316.25 mM NaCl, and 0.006% DDM. Reactions were run for 6 hours at 25°C, quenched by incubation at 98°C for 5 minutes, and cooled to room temperature. Biotinylated-D-lysine (2 mM, working concentration) and purified *E. faecalis* PBPX (10 µM, working concentration) were added to the reactions and incubated at 25°C for 30 minutes to biotinylate the peptidoglycan products. The reaction was quenched by the addition of 6.5 µL 5× Laemmli sample buffer. 26 µL was loaded on 4%–20% gradient polyacrylamide gel (BioRad) and subjected to electrophoresis at 180 volts for 35 minutes. The gels were then transferred to PVDF (BioRad), fixed with 0.4% paraformaldehyde in PBS for 30 minutes, and blocked with SuperBlock (Thermo Fisher Scientific) for 1 hour at room temperature. Membranes were probed with IRDye 800CW Streptavidin (LI-COR Biosciences) diluted 1:5,000 in SuperBlock for 1 hour at room temperature, washed three times with PBS, and visualized with Amersham Typhoon Imager (GE Life Sciences).

### Antimicrobial susceptibility assays and growth curves

Stationary-phase cultures were normalized to OD_600_ = 4 × 10^−5^ (~1 × 10^5^ CFU) and inoculated into 100-well honeycomb plates containing twofold serial dilutions of ceftriaxone (1,024–4 µg/mL) and antibiotic for plasmid maintenance as appropriate. Cultures were supplemented with 10 µM staurosporine or DMSO vehicle control to determine the impact of IreK inhibition on MIC. Cultures were grown for 24 hours at 37°C in a Bioscreen C plate reader. Growth was monitored at OD_600_, every 15 minutes, with shaking before each measurement. The MIC was determined as the lowest concentration of ceftriaxone to inhibit growth over 24 hours. Growth curves were obtained from the samples prepared in the same manner, grown in the absence of ceftriaxone.

### Cell wall integrity assessment

Cell wall integrity was assessed by chlorophenol red-β-d-galactopyranoside (CPRG) hydrolysis as previously described by Djoric et al. (53). Briefly, cultures supplemented with 10 µg/mL erythromycin (for maintenance of pCJK205, constitutively expressing β-galactosidase) were grown to stationary phase in the presence of 40 µg/mL CPRG. CPRG hydrolysis was measured at OD_570_, normalized to bacterial growth at OD_630_. We previously demonstrated that this method could detect changes to cell wall integrity as subinhibitory concentrations of cell wall active antibiotics (bacitracin, ceftriaxone) induced CPRG hydrolysis, while kanamycin did not (53).

### Whole-cell lysate preparation

Stationary-phase cultures were normalized to OD_600_ = 0.01 in MHB (supplemented with 10 µg/mL chloramphenicol when required for maintenance of plasmids) and grown to exponential phase, shaking at 37°C. Cells were harvested by the addition of an equal volume of ice-cold ethanol/acetone (1:1) and collected by centrifugation at 4,000 rpm 4°C, followed by washing with 1 mL water and resuspension in lysozyme solution [10 mM Tris, 20% sucrose, 50 mM NaCl (pH 8.0)]. Cells were normalized by OD_600_, treated with 5 mg/mL lysozyme for 20 minutes at 37°C, and mixed with 5× non-reducing Laemmli buffer. Cell lysates used for phos-tag SDS-PAGE were not boiled, but lysates for standard immunoblot were boiled for 5 minutes. Cell lysates were stored at −20°C.

### Standard SDS-PAGE and immunoblotting

Whole-cell lysates were separated by SDS-PAGE, supplemented with 0.5% 2,2,2-trichloroethanol (TCE) for stain-free total protein visualization, at 150 V for 1 hour, followed by UV activation of TCE using a ChemiDoc imaging system (BioRad) and transfer to polyvinylidene fluoride (PVDF) membrane (BioRad) using a Transblot Turbo system (BioRad) at 25 V for 14 minutes. After transfer, TCE was imaged using a ChemiDoc imaging system, and membranes were blocked in 5% milk for 1 hour at room temperature. Membranes were probed with custom primary antiserum to detect MltG or 6xhis tag polyclonal antibody (Invitrogen) to detect MltG-his, each diluted 1:5,000 in TBS-T. Membranes were then probed with horseradish peroxidase-conjugated secondary antibody, diluted 1:5,000 in 5% milk, and imaged using a ChemiDoc imaging system. Image Lab software (BioRad) was used for protein quantitation.

### Phos-tag SDS-PAGE

Immunoblot analysis for phos-tag SDS-PAGE of IreK was performed as previously described by Minton et al. (41), and immunoblot analysis for phos-tag SDS-PAGE of CroR was performed as previously described by Kellogg et al. (31). Briefly, whole-cell lysates were separated on 6% SDS-PAGE gels supplemented with 50 µM Phos-tag and 100 µM Zn(NO_3_)_2_ (IreK phos-tag gels) or 10% SDS-PAGE gels supplemented with 20 µM Phos-tag and 40 µM MnCl_2_ (CroR phos-tag gels). Electrophoresis conditions were 200 V for 50 min (at 4°C for CroR). Following electrophoresis, all Phos-tag gels were soaked in 5 mM EDTA 3 times for 10 min each. Proteins were transferred to nitrocellulose membranes (for IreK) or PVDF (for CroR) using a Transblot Turbo system at 25 V for 14 minutes. Membranes were probed with custom primary antisera to detect IreK or CroR.

### Rate of PG synthesis

Peptidoglycan synthesis assays were performed as described by Mascari et al. (23) Briefly, cultures were grown as described above and diluted into pre-warmed MHB media supplemented with 0.33 mCi/mL [^14^C] GlcNAc or label-free media. Incorporation of [^14^C] GlcNAc into the insoluble cell wall fraction was monitored at regular time intervals by mixing with 0.2% SDS, washing with water, and transferring to Econo-safe scintillation fluid for scintillation counting. Culture growth was monitored by measuring the OD_600_ of label-free samples using a SpectraMax5 plate reader (Molecular Devices).

### Bocillin-FL PBP labeling and expression

*In vivo* labeling of penicillin binding proteins (PBPs) was assessed by Bocillin-FL labeling as previously described by Djoric et al. (21). Bacteria were grown to the exponential phase in MHB at 37°C as described above. Cells were then chilled on ice, collected by centrifugation (8,000 rpm for 10 minutes at 4°C), and resuspended in Bocillin labeling buffer (25 mM Tris [pH 7.5], 150 mM NaCl, 200 µg/mL chloramphenicol). Cells were normalized to OD_600_ = 30. Bocillin FL was added to a final concentration of 50 µM, and samples were incubated for 20 minutes at 37°C. Cells were then collected by centrifugation, washed with Bocillin labeling buffer, resuspended in 100 mg/mL lysozyme in lysozyme solution, and incubated at 37°C for 15 minutes. After the incubation, Laemmli SDS sample buffer was added, and samples were boiled for 5 minutes. Samples were subjected to 8% SDS-PAGE on 20 cm gels at constant amperage for ∼16 hours and visualized with Amersham Typhoon Imager (GE Life Sciences). After fluorescence images were acquired, GelCode Blue was used to stain for total protein. To determine whether increased PBP labeling was due to a change in expression, samples were subjected to SDS-PAGE as described above and probed with custom primary antisera to detect PbpA and Pbp4.

## SUPPLEMENTAL MATERIAL

Supplemental material jb.00056-25-s0001.pdf Fig. S1 to S4, Tables S1 and S2, and Supplemental references.

ASM does not own the copyrights to Supplemental Material that may be linked to, or accessed through, an article. The authors have granted ASM a non-exclusive, world-wide license to publish the Supplemental Material files. Please contact the corresponding author directly for reuse.
