# Supplementary material for: The cell wall hydrolase MltG is essential to maintain cell wall homeostasis of Enterococcus faecalis
Source: J Bacteriol. 2025 Jun 13;207(7):e00056-25. doi: 10.1128/jb.00056-25 (PMC12288455; doi:10.1128/jb.00056-25)
Supplement: Supplemental material — Fig. S1 to S4, Tables S1 and S2, and Supplemental references. [file jb.00056-25-s0001.pdf]

Supplemental Material for:

Title: The cell wall hydrolase MltG is essential to maintain cell wall homeostasis of *Enterococcus faecalis*

Authors: Alexis AU Knotek, Christopher J Kristich[#]

Running title: MltG maintains cell wall homeostasis

Affiliation:

Department of Microbiology and Immunology

Center for Infectious Disease Research

Medical College of Wisconsin

8701 Watertown Plank Rd

Milwaukee, WI 53226

*For correspondence: ckristich@mcw.edu

```
CLUSTAL O(1.2.4) multiple sequence alignment

Ec      ------------------------------------------------------------ 0
Bs      ------------------------------------------------------------ 0
Sp      MSEKSREEEKLSFKEQILRDLEKVKGYDEVLKEDEAVVRTPANEPSAEELMADSLSTVEE 60
WT      ------------------------------------------------------------ 0
Ef      ------------------------------------------------------------ 0

Ec      ------------------------------------------------------------ 0
Bs      ------------------------------------------------------------ 0
Sp      IMRKAPTVPTHPSQGVPASPADEIQRETPGV-PSHPSQDVPSSPAEESGSRPGPGPVRPK 119
WT      -MANDNQNNQD--------PKSSLRDQVTGSLKGRNDGDQPDSS-EKNDRSP--QPSSD- 47
Ef      ------------------------------------------------------------ 0

Ec      ------------------------------------------------------------ 0
Bs      ------------------------------------------------------------ 0
Sp      KLEREYNETPTRVAVSYTT---A---EKKAEQAGPETPTPATETVDIIRDTSRRSRREGA 173
WT      -----ESQETASRTTQTRAGSRAARRRGKDKTTQTVEEPTPIETDEKPTN-TKKQ----- 96
Ef      ----------------MSGNKQHP--SKDKKE------ASKAATADIID-KRRN------ 29

Ec      -----------MKKVLLIIILLLLVVLGIAAGVGVWKVRHLA--DSKLLIKEETIFTLKPG 47
Bs      --MIENQVKKKRRRIFLFSIIALLLVC----GSVYAYISSALGPVDSGNKKEIEVEIPKG 54
Sp      KPAKPKKEKKSHVKAFVISFLVFLALLSAGGYFGYQYVLDSLLPIDANSKKYVTVGIPEG 233
WT      ---TRKKEDRLVGRIVLIVVSVLVLMMAIFGFTFYKYVDAGLQPLDKNNKKLVQVHIPEG 153
Ef      ---LRKKEDKIVRKIILVIALTLLIIGGFLGFTVYRYVDSGLKPLDKSDDQLVQVEIPSG 86
                      : .:.    :: :          :     .    .   .:    . :  *
Ec      TGRLALGEQLYADKIINRPRVFQWLLRIEPDLSHFKAGTYRFTPQMTVREMLKLLESGKE 107
Bs      SSTSKIGEILEEKGAVKNGTVFSFYTKA-KS-KNLQAGTYLLNPSMNAKDVIEQMSSGNV 112
Sp      SNVQEIGTTLEKAGLVKHGLIFSFYAKY-KNYTDLKAGYYNLQKSMSTEDLLKELQKGGT 292
WT      SSNKQIAAVLEESNVIKSGMVFNYYVKF-KNLTDFQAGYYQMSPSMTLDEIGEMLKEGGT 212
Ef      SSNKQIGEILEKDNIIKSGIVFNYYTKF-KNLTGFQAGYYQLAPNMTLDEIGKQLQEGGT 145
        :.   :.  *     ::   :*.:  :   . . ::** * :  .*.  :: : :..*
Ec      AQ------FPLRLVEGMRLS--------------------DYLKQLREAPYIKHTLSDDK 141
Bs      HR---PALYKVTIKEGAQVTEIAETIAAELKW-----NKDDVTRQLNDKAFIQKMQ--QK 162
Sp      DEPQEPVLATLTIPEGYTLDQIAQTVGQLQGDFKESLTAEAFLAKVQDETFISQAV--AK 350
WT      PEPTKIADGKVTIPEGYDIDKIGEAIEKNTDF-----KKADFIALMKNEDFFNQMK--AK 265
Ef      SEPTKVADGKIAIPEGYDIDQIAERVAKVTGK-----DKKEFLDLVNDETFFNELH--QK 198
         .         : : **  :                          :.:  ::..      *
Ec      YATVAQAL---ELENPEWIEGWFWPDTWMYTANTTDVALLKRAHKKMVKAVDSAWEGRAD 198
Bs      YPKLLTDKI-FDSNIKYPLEGYLYPATYSFYKKDTTLEEI---VIPMLEKTNAIIVQNEA 218
Sp      YPTLLESLPVKDSGARYRLEGYLFPATYSIKES-TTIESL---IDEMLAAMDKNLSPYYS 406
WT      YPDLLESAA-TAEGVRYRLEGYLFPATYDYYKK-ATLPEF---VEQMIAKMNTVMEQYTP 320
Ef      FPELLESAS-KAENTKYRLEGYLFPATYDYYTS-TSLKDL---VIEMVNKTNTVMQNYYS 253
        :  :                :**:::* *:     . : :  :       *:   :
Ec      GL--PYKDKNQLVTMASIIEKETAVASERDKVASVFINRLRIGMRLQTDPTVIYGMGERY 256
Bs      KMKAKNWDVHQLLTLSSLIEEEATGFTDRQKISSVFYNRLEKGMPLQTDPTVLYALGKHK 278
Sp      TIKSKNLTVNELLTIASLVEKEGAKTEDRKLIAGVFYNRLNRDMPLQSNIAILYAQGKLG 466
WT      TIHAKNLTNQQVLTLASLVEKEGVKEADRKQIAQVFFNRLAADMPIQSDISILYALGEHK 380
Ef      AIKQKNLTVQEVLTLASLVEKEGVKENDRKNIAQVFFNRIKANMPLQSDISVLYALGEHK 313
         :         ::::*::*::*:* .    :*. :: ** **:  .* :*:: :::*. *:
Ec      NGKLS----RADLETPTAYNTYTITGLPPGAIATPGADSLKAAAHPAKTPYLYFVADGKG 312
Bs      QRVLY-----EDLKVNSPYNTYVVKGLPVGPIANSGKHSVEAALEPAQTDYYYFLAA-PS 332
Sp      QNISLAEDVAIDTNIDSPYNVYKNVGLMPGPVDSPSLDAIESSINQTKSDNLYFVADVTE 526
WT      ETVTY-----ADLEVDSSYNLYKNTGYGPGPLDSPSEESIKAVLNPTPSDYLYFVADIST 435
Ef      ELVTY-----EDTAVDSPYNLYTNTGYGPGPFDNPSEEAIKAVLEPAENDYYYFVADTST 368
        :           *     : ** *    *    *. .  . .::::  . : .    **:*
Ec      GH-TFNTNLASHNKSVQDYLKVLKEKNAQ      340
Bs      GEVYYAKTLEEHNALKQKYITKKQ-----      356
Sp      GKVYYANNQEDHDRNVAEHVNSKLN----      551
WT      GKVYFSKTYEEHQVLVDQYVNNSSSE---      461
Ef      GNVYFAKTYEEHMELVEKYVNNS------      391
        *.  : ..   .*     .::.
```

Figure S1. MltG homolog sequence alignment. Amino acid sequence alignment of *E. faecalis* (WT) MltG (OGIRF_12215) with *Escherichia coli* (Ec) MltG (QPA14937.1), *Bacillus subtilis* (Bs) MltG (UOC03447.1), *Streptococcus pneumoniae* (Sp) MltG (AVN86126.1), and *E. faecium* (Ef) MltG (WP_002289159.1). LysM domain of WT MltG highlighted in yellow. YceG domain of WT MltG highlighted in light green, with conserved catalytic glutamate residue highlighted in dark green.

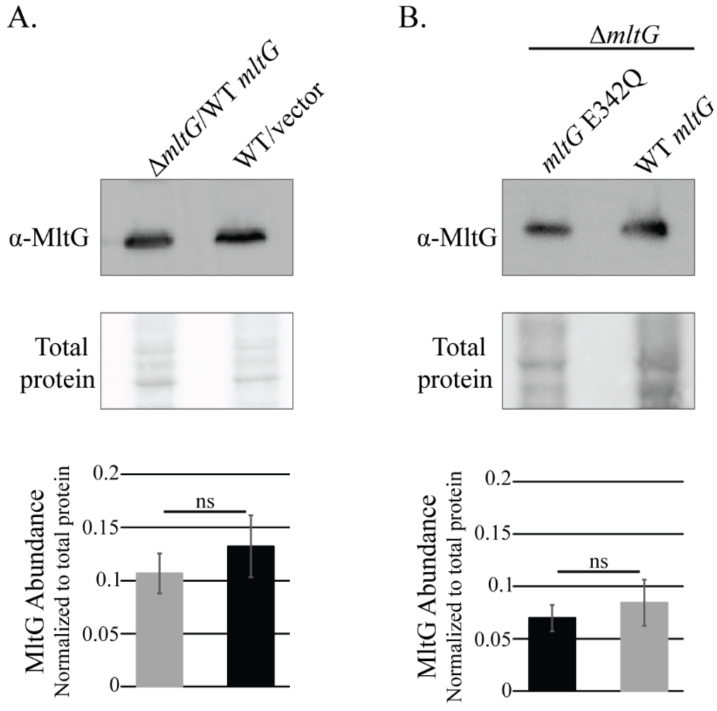

Figure S2. Ectopic expression of WT MltG and MltG E342Q. Ectopic expression of MltG from plasmids was confirmed by subjecting whole cell lysates to SDS-PAGE supplemented with TCE for total protein detection and immunoblot with MltG antiserum. A) WT *mltG* is expressed from a plasmid at the same level as *mltG* is expressed from the WT/vector chromosome. B) Expression of MltG E342Q mutant was compared to that of WT *mltG* expressed form the same plasmid. n=3 and error bars represent +/- s.d. * = $p<0.05$; ns = not significant. Student's t-test(heteroschidastic, two-tailed). Strains were WT/vector = OG1(pJRG9); Δ*mltG/*WT *mltG* = JL650(pAAU12); Δ*mltG*/*mltG* E342Q = JL650(pAAK15).

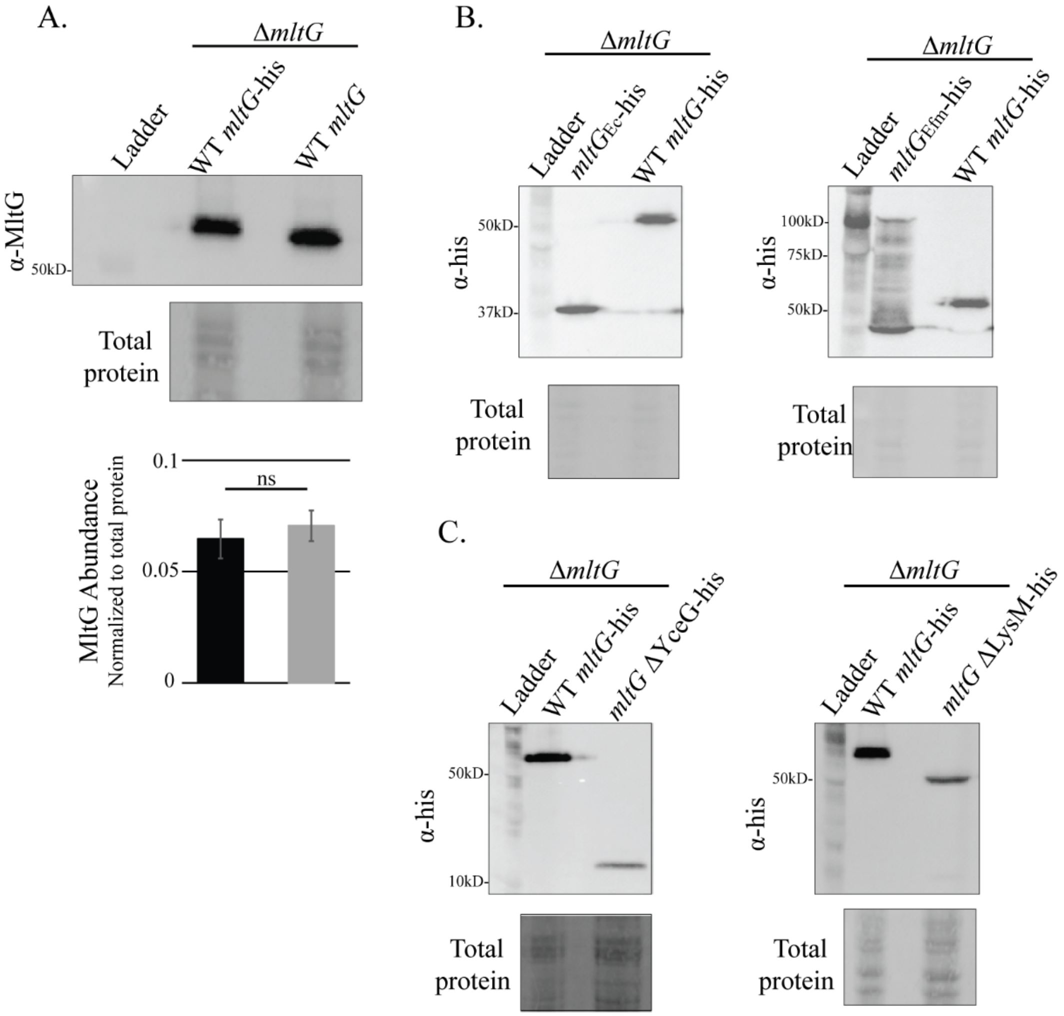

Figure S3. Expression of His-tagged MltG homologs and truncation mutants. Ectopic expression of MltG-his from plasmids was confirmed by subjecting whole cell lysates to SDS-PAGE supplemented with TCE for total protein detection and immunoblot with MltG or his$_6$ antiserum. A) WT *mltG*-his is stably expressed from a plasmid, as seen for WT *mltG*. B) The MltG *E. coli* homolog (MltG$_{Ec}$-his) and *E. faecium* MltG homolog (MltG$_{Efm}$-his) are stably expressed. C) MltG-his ΔYceG and ΔLysM truncation mutants are stably expressed. Strains were Δ*mltG/*WT *mltG* = JL650(pAAU12); Δ*mltG* /WT *mltG-his* = JL650(pAAK33); Δ*mltG/mltG*$_{Ec}$ = JL650(pAAK75); Δ*mltG/mltG*$_{Efm}$ = JL650(pAAK76); Δ*mltG*/*mltG-his* ΔYceG = JL650(pAAK66); Δ*mltG/mltG-his* ΔLysM = JL650(pAAK68).

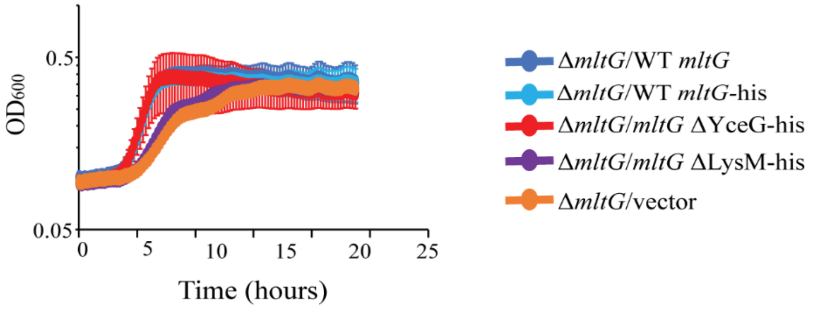

Figure S4. Growth curve of His-tagged MltG truncation mutants. Bacterial growth, measured as OD_{600}, was monitored over the course of 24 hours. Data represent the mean ± standard deviation of three biological replicates. Strains were Δ*mltG/*WT *mltG* = JL650(pAAU12); Δ*mltG/*vector= JL650(pJRG9); Δ*mltG/*WT *mltG*-his = JL650(pAAK33); Δ*mltG/mltG*-his ΔYceG = JL650(pAAK66); Δ*mltG/mltG* ΔLsyM = JL650(pAAK68).

Table S1. Ceftriaxone resistance of His-tagged MltG truncation mutants.

Strain[b]	Ceftriaxone MIC (μg/mL)[a]
Δ*mltG*/vector	512
Δ*mltG*/WT *mltG*	64
Δ*mltG*/*mltG* ΔYceG	256
Δ*mltG*/*mltG* ΔLysM	512

[a]The median MIC of ceftriaxone determined from at least 3 biological replicates

[b]Strains were Δ*mltG/*vector= JL650(pJRG9); Δ*mltG/*WT *mltG* = JL650(pAAU12); Δ*mltG/mltG* ΔYceG = JL650(pAAK65); Δ*mltG/mltG* ΔLsyM = JL650(pAAK67).

Table S2. Strains and plasmids used in this study

Strain or plasmid	Description of genotype	Source or reference
**STRAINS**		
E. coli		
TOP10	Routine cloning host	Lab stock
DH5α	Routine cloning host	Lab stock
BL21(DE3)	Protein overexpression host	Lab stock
C43(DE3)	Protein overexpression host	Lucigen
Nico21 (DE3)	Protein overexpression host	NEB
E. faecalis		
OG1	Wild-type, original unmarked isolate	Gold 1975[1]
OG1RF	Spontaneous rifampicin- and fusidic acid-resistant derivative of OG1	Dunny 1978[2]

JL650	OG1 Δ*mltG* (ΔQ6-D452)	Minton 2022[3]
JL206	OG1 Δ*ireK*	Labbe 2017[4]
SB23	OG1 Δ*croR2*	Snyder 2014[5]
JL339	OG1 Δ*pbp4* (ΔS11-A677)	Kristich 2012[6]
JL632	OG1 Δ*pbpA* (ΔF6-E707)	Djoric 2020[7]
AK6	OG1 dep-*mltG* Δ*croR*	This work
JL704	OG1 Δ*pbp4* Δ*mltG* (ΔS11-A677;ΔQ6-D452)	This work
**PLASMIDS**		
pJH086	*E. faecalis* allelic-exchange vector (Cm^r, repA V71G, lacZ, pheS*)	Kellogg 2017[8]
pJLL272	Δ$mltG_{Efs}$ deletion allele in pJH086	Minton 2022[3]
pET28a::his_6-smt3	his_6-smt3 *E. coli* protein expression vector (Kn^r) with SUMO cleavable his_6 tag	Brian Volkman Lab
pAAK42	pET28a::his_6-smt3-*mltG*	This work
pAAK55	pET28a::his_6-smt3-*mltG* E342Q	This work
pET28b	*E. coli* protein expression vector (Kn^r)	Novagen
pLMM25	pET28b-his_6-*pbpX* (T36-P429)	Nelson 2024[9]
pMEL66	pET28b::ΔTM *Pbp1a*-His (Sp; Δ1-36)	This work
pJRG9	*E. faecalis* expression vector, constitutive P23s promoter (Cm^r)	Snyder 2014[5]
pAAU12	pJRG9::WT *mltG*	This work
pAAK15	pJRG9::*mltG* E342Q	This work

pAAK67	pJRG9::*mltG* ΔLysM (ΔN141-P215)	This work
pAAK68	pJRG9::*mltG*-his$_6$ ΔLysM (ΔN141-P215)	This work
pAAK65	pJRG9::*mltG* ΔYceG (ΔA220-E461)	This work
pAAK66	pJRG9::*mltG*-his$_6$ ΔYceG (ΔA220-E461)	This work
pAAK75	pJRG9::*mltG*$_{Ec}$-his$_6$	This work
pAAK76	pJRG9::*mltG*$_{Efm}$-his$_6$	This work
pJLL286	*E. faecalis* nitrate inducible expression vector (Em^{r})	Mascari 2022[10]
pNPC1	pJLL286::WT *mltG*	This work
pJLL371	pJLL286::WT *pbp4*	This work

Supplemental References

1. Gold OG, Jordan HV, van Houte J. The prevalence of enterococci in the human mouth and their pathogenicity in animal models. *Arch Oral Biol*. 1975;20(7):473-IN15. doi:10.1016/0003-9969(75)90236-8

2. Dunny GM, Brown BL, Clewell DB. Induced cell aggregation and mating in Streptococcus faecalis: evidence for a bacterial sex pheromone. *Proceedings of the National Academy of Sciences*. 1978;75(7):3479-3483. doi:10.1073/pnas.75.7.3479

3. Minton N, Djoric D, Little J, Kristich CJ. GpsB promotes PASTA kinase signaling and cephalosporin resistance in Enterococcus faecalis. *J Bacteriol*. 2022;204(10). doi:https://doi.org/10.1128/jb.00304-22

4. Labbe BD, Kristich CJ. *Growth-and Stress-Induced PASTA Kinase Phosphorylation in Enterococcus Faecalis*.; 2017. https://doi.org/10

5. Snyder H, Kellogg SL, Skarda LM, Little JL, Kristich CJ. Nutritional control of antibiotic resistance via an interface between the phosphotransferase system and a two-component signaling system. *Antimicrob Agents Chemother*. 2014;58(2):957-965. doi:10.1128/AAC.01919-13

6. Kristich CJ, Little JL. Mutations in the β subunit of RNA polymerase alter intrinsic cephalosporin resistance in enterococci. *Antimicrob Agents Chemother*. 2012;56(4):2022-2027. doi:10.1128/AAC.06077-11

7. Djorić D, Little JL, Kristich CJ. Multiple low-reactivity class B penicillin-binding proteins are required for cephalosporin resistance in enterococci. *Antimicrob Agents Chemother*. 2020;64(4). doi:10.1128/AAC.02273-19

8. Kellogg SL, Little JL, Hoff JS, Kristich CJ. Requirement of the CroRS two-component system for resistance to cell wall-targeting antimicrobials in Enterococcus faecium. *Antimicrob Agents Chemother*. 2017;61(5). doi:10.1128/AAC.02461-16

9. Nelson ME, Little JL, Kristich CJ. Pbp4 provides transpeptidase activity to the FtsW-PbpB peptidoglycan synthase to drive cephalosporin resistance in Enterococcus faecalis. *Antimicrob Agents Chemother*. 2024;68(9):e0055524. doi:10.1128/aac.00555-24

10. Mascari CA, Djorić D, Little JL, Kristich CJ. Use of an Interspecies Chimeric Receptor for Inducible Gene Expression Reveals that Metabolic Flux through the Peptidoglycan Biosynthesis Pathway is an Important Driver of Cephalosporin Resistance in Enterococcus faecalis. *J Bacteriol*. 2022;204(4). doi:10.1128/jb.00602-21
